# Complete mitochondrial DNA sequence of the parasitic honey bee mite *Varroa destructor* (Mesostigmata: Varroidae)

**DOI:** 10.1080/23802359.2019.1711219

**Published:** 2020-01-16

**Authors:** Reona Harada, Masato Yoshioka, Hisashi Okuyama, Manabu Kato, Stephen J. Martin, Jun-ichi Takahashi

**Affiliations:** a Faculty of Life Sciences, Kyoto Sangyo University, Kyoto, Japan;; b Yamada Bee Company Inc., Tomata-gun, Japan;; c School of Environment and Life Sciences, The University of Salford, Manchester, UK

**Keywords:** *Varroa* disease, colony collapse, apiculture, beekeeping, honey bee

## Abstract

*Varroa destructor* is a parasite mite of the eastern honey bee *Apis cerana*, which is native to Asia. The European honey bee *Apis mellifera* was imported to Asia from Europe and the USA for apiculture in the 19th century. In a short period of time, *V. destructor* parasitized the artificially introduced honey bees. *Varroa destructor* was estimated to have spread around the world with *A. mellifera* when it was exported from Asia to locations worldwide about 50 years ago. The mitochondrial DNA of the parasitic honey bee mite *V. destructor* was analyzed using next-generation sequencing. The complete mitochondrial genome of *V. destructor* was identified as a 16,476-bp circular molecule containing 13 protein-coding genes (PCGs), 22 tRNA genes, two rRNA genes, and one AT-rich control region. The heavy strand was predicted to have nine PCGs and 13 tRNA genes, whereas the light strand was predicted to contain four PCGs, nine tRNA genes, and two rRNA genes. All PCGs began with ATA as the start codon, except *COIII* and *CytB*, which had ATG as the start codon. Stop codons were of two types: TAA for eight genes and TAG for five genes. Molecular phylogenetic analysis revealed that *V. destructor* from Japan was genetically distant from that of France. A high base substitution rate of 2.82% was also confirmed between the complete mitochondrial DNA sequences of *V. destructor* from Japan and the USA, suggesting that one *Varroa* mite strain found in the USA is not from Japan.

The mite *Varroa destructor* is a parasite of the eastern honey bee *Apis cerana*, which is native to Asia (Anderson 2000). The European honey bee *Apis mellifera* was imported to Asia from Europe and the USA for apiculture in the 19th century. In a short period of time, *V. destructor* parasitized the artificially introduced honey bees (Anderson and Trueman 2000). *Varroa destructor* was estimated to have spread around the world with *A. mellifera* when it was exported from Asia to Europe, the Americas, and Africa about 50 years ago (Ellis et al. 2010). Because *A. mellifera* is not resistant to this mite, a honey bee colony infected with *Varroa* mites eventually collapses. In the USA, *Varroa* disease caused by *V. destructor* is recognized as the most serious disease and the main cause of death among *A. mellifera* colonies (Martin et al. 2012; Martin 2013). Mitochondrial DNA sequence data can provide useful information to estimate the invasion route of *Varroa* mites (Evans and Lopez 2002; Navajas et al. 2002). Therefore, we determined the complete mitochondrial genome of the native *Varroa* mite in Japan.

We collected several *V. destructor* adults from an *A. mellifera* hive from an apiary in Kagamino-cho, Okayama Prefecture, Japan, in July 2014 (35°07′05.2″N 133°54′23.7″E). The specimen was stored in a freezer at −20 °C in National Museum of Nature and Science, Japan . The adult mites were transferred immediately to 99% ethanol for subsequent mitochondrial DNA analysis. To sequence the mitochondrial DNA, we used MiSeq (Illumina). These specimens were stored at the National Museum of Nature and Science, Japan. The complete mitochondrial genome of *V. destructor* from the USA was used as a reference sequence to assemble the reads using Geneious R9 (Bernt et al. 2013). The complete draft of the mitochondrial DNA sequence was annotated using the MITOS web server (Kearse et al. 2012). The identified tRNA genes were verified using the tRNAscan-SE program (Lowe and Eddy 1997). The AT content and codon usage were calculated using Geneious R9. The phylogenetic analysis was performed under the maximum likelihood (ML) criterion using TREEFINDER (Jobb 2011).

We succeeded in sequencing the entire mitochondrial genome of *V. destructor* from Japan (GenBank under the accession number AP019523). The genome consisted of a 16,476-bp long closed loop, which included 13 protein-coding genes (PCGs), 22 tRNA genes, two rRNA genes, and one AT-rich control region that represents a typical mite mitochondrial genome. The average AT content of the *V. destructor* mitochondrial genome was 80.1%. The heavy strand was predicted to have nine PCGs and 13 tRNA genes, whereas the light strand was predicted to contain four PCGs, nine tRNA genes, and two rRNA genes. All PCGs began with ATA as the start codon, except *COIII* and *CytB*, which had ATG as the start codon. Stop codons were of two types: TAA for eight genes (*ND1*, *ND2, COIII, ATP6*, *ATP8*, *ND4*, *ND4L*, and *ND6*) and TAG for five genes (*COI*, *COII*, *ND3*, *ND5*, and *CytB*). A phylogenetic analysis was performed using 13 PCGs across 14 Acari taxa (Figure 1). A high base substitution rate of 2.82% (464/16476) was confirmed between the complete mitochondrial DNA sequences of *V. destructor* from Japan and the USA, suggesting that one *Varroa* mite strain found in the USA is not from Japan.

**Figure 1.**
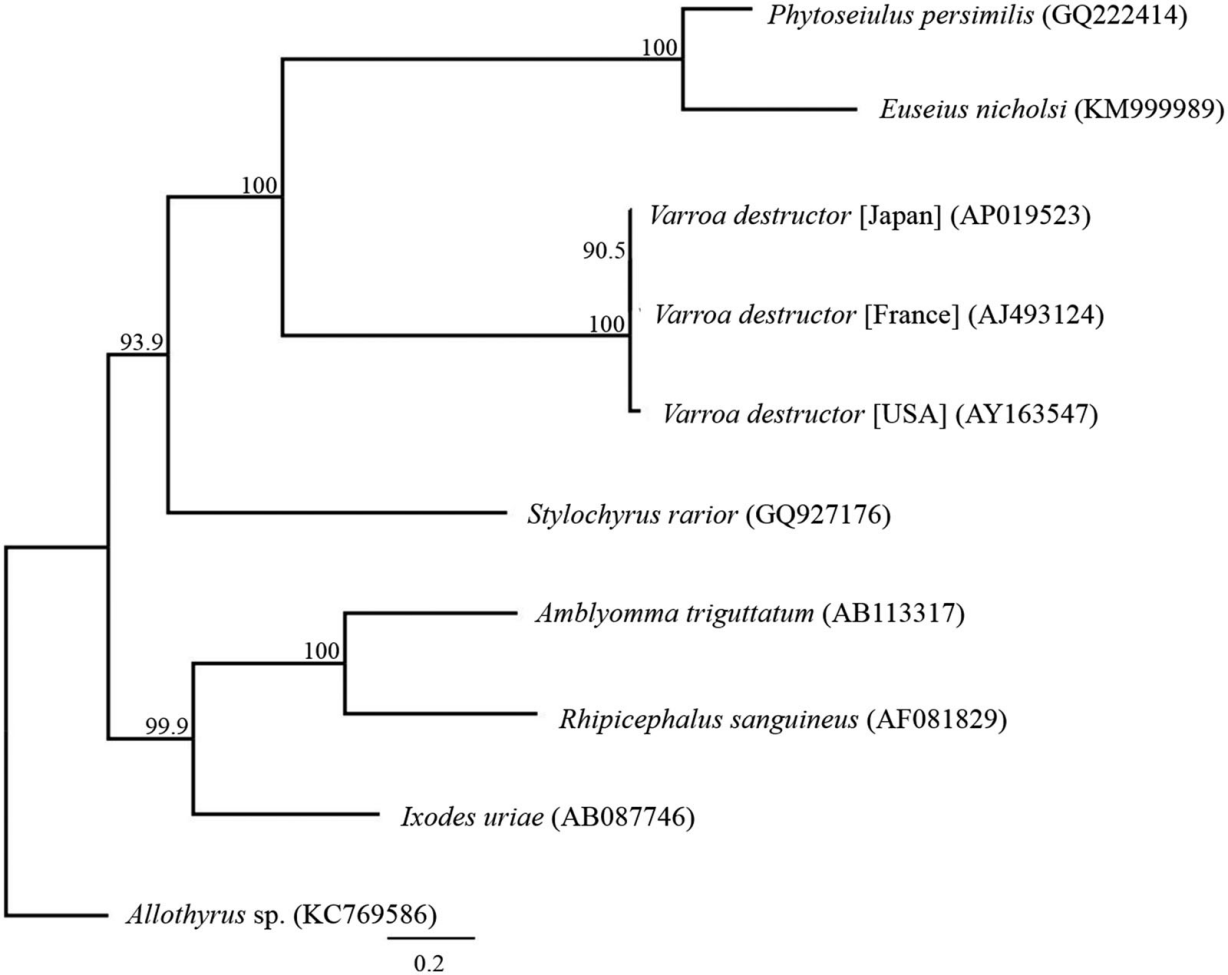
Phylogenetic relationships (maximum-likelihood) of the Mesostigmata based on the nucleotide sequences of the 13 protein-coding genes of the mitochondrial genome. The numbers at the nodes indicate the bootstrap support inferred from 1000 bootstrap replicates. Alphanumeric terms indicate the DNA Database of Japan accession numbers.
